# Dopant size effects on novel functionalities: High-temperature interfacial superconductivity

**DOI:** 10.1038/s41598-017-00539-4

**Published:** 2017-03-28

**Authors:** Y. Eren Suyolcu, Yi Wang, Federico Baiutti, Ameer Al-Temimy, Giuliano Gregori, Georg Cristiani, Wilfried Sigle, Joachim Maier, Peter A. van Aken, Gennady Logvenov

**Affiliations:** 1 Max Planck Institute for Solid State Research, Heisenbergstrasse 1, 70569 Stuttgart, Germany; 2 Al-Nahrain Nanorenewable Energy Research Center, Al-Nahrain University, Baghdad, Iraq

## Abstract

Among the range of complex interactions, especially at the interfaces of epitaxial oxide systems, contributing to the occurrence of intriguing effects, a predominant role is played by the local structural parameters. In this study, oxide molecular beam epitaxy grown lanthanum cuprate-based bilayers (consisting of a metallic (M) and an insulating phase (I)), in which high-temperature superconductivity arises as a consequence of interface effects, are considered. With the aim of assessing the role of the dopant size on local crystal structure and chemistry, and on the interface functionalities, different dopants (Ca^2+^, Sr^2+^ and, Ba^2+^) are employed in the M-phase, and the M–I bilayers are investigated by complementary techniques, including spherical-aberration-corrected scanning transmission electron microscopy. A series of exciting outcomes are found: (i) the average out-of-plane lattice parameter of the bilayers is linearly dependent on the dopant ion size, (ii) each dopant redistributes at the interface with a characteristic diffusion length, and (iii) the superconductivity properties are highly dependent on the dopant of choice. Hence, this study highlights the profound impact of the dopant size and related interface chemistry on the functionalities of superconducting oxide systems.

## Introduction

High-quality complex oxide heterostructures are excellent systems for studying interface phenomena arising from the interaction between neighboring layers^1, 2^. Depending on the choice of the constituents, different microscopic phenomena can occur at the interfaces, including e.g. electronic and orbital reconstruction, magnetic exchange interactions, crystal-structure distortions, chemical intermixing, or breaking of the crystal symmetry^3–9^.

In this context, one recent exciting finding was the observation by Gozar *et al*. of high-temperature interface superconductivity (HT-IS) at the interface between epitaxially grown strontium-overdoped metallic (M) lanthanum cuprate (La_1.55_Sr_0.45_CuO_4_) and underdoped insulating (I) La_2_CuO_4_ (LCO) layers^10^, none of which is superconducting if taken alone. The full understanding of HT-IS is a very important step towards the disclosure of the mechanism of high-temperature superconductivity (HTS)^10, 11^, being potentially able to shed light on questions related to the formation of superconducting interfaces^10, 12^, its dimensionality and locus^13^ and the impact of the crystal structure and atom positions on the superconducting properties^14, 15^. Numerous studies employing advanced experimental methods as well as innovative approaches have addressed these questions^13, 14, 16–21^ and the state-of-the-art of the common understanding of such phenomena can be found in several review articles^22–28^.

In order to explain the HT-IS in M–I lanthanum cuprate bilayers, a model based on electronic charge transfer due to the difference in the hole chemical potentials between the overdoped and the underdoped phases has been invoked^13, 20^. As a consequence of such a redistribution, a doped region having optimal hole concentration for HTS (namely, the second CuO_2_ plane in LCO away from the interface) is formed in the nominally insulating phase. Notably, in such bilayers, the superconducting critical temperature (*T*_c_) was also found to be dependent on the deposition sequence, being about 15 K in I–M structures (in which the LCO layer is deposited first) and >30 K in M–I structures, in which overdoped LSCO is employed as a bottom layer^14^. Remarkably, despite the two phases taken singularly exhibit quite different *c*-axis parameter (~13.25 Å and ~13.15 Å for LSCO and LCO, respectively), a common *c* can be assigned to both phases, according to X-ray diffraction (XRD) analysis, in the case of epitaxial bilayers. Namely, the top layer adopts the out-of-plane lattice parameter of the bottom phase as a consequence of electrostatic interactions (“Madelung strain”)^14^. The dependence of *T*_c_ on the deposition sequence was attributed to such an effect, with *T*_c_ being higher (lower) when the out-of-plane lattice constant of the structure is expanded (shrunk) due to Madelung strain (i.e. when M is deposited first). Notably, a linear relation between *T*_c_ and *c* was pointed out^14^.

Such findings open an exciting scenario for the enhancement of the superconducting critical temperature in M–I lanthanum cuprate heterostructures, which could in principle be obtained by appropriately tuning the out-of-plane lattice parameter of the bottom layer. In particular, a promising route is represented by the possibility of realizing overdoped La_2−*x*_Ba_*x*_CuO_4+δ_ (LBCO) thin films, as demonstrated by Sato *et al*.^29^, which was found to have *c* as high as ~13.5 Å for strongly overdoped (*x* = 0.35) layers under compressive strain (grown on LaSrAlO_4_ (001) substrates). In the light of these considerations, the extrapolated *T*_c_ value for the M-I bilayer with LBCO being the bottom layer could lead to a giant enhancement up to ~70 K.

In this work, we investigated La_1.6_A_0.4_CuO_4_–La_2_CuO_4_ bilayers (with A = Ca, Sr, Ba), which were grown by using the atomic-layer-by-layer oxide molecular beam epitaxy technique (ALL-oxide MBE)^30^, by employing several and complementary experimental techniques: atomic force microscopy (AFM), XRD, low-temperature direct current (DC) resistance measurements, magnetic susceptibility measurements and high-resolution scanning transmission electron microscopy (STEM). It should be noted here that, whereas the successful synthesis of high-quality Sr-doped^31^ and Ba-doped^29^ La_2_CuO_4_ epitaxial films have been achieved by several groups, there are no reports about MBE-grown Ca-doped LCO epitaxial films, possibly as a consequence of low Ca solubility^32^ or due to the tendency of the formation of a highly defective structure (high concentration of oxygen vacancies)^33^. Note also that Ca^2+^, Sr^2+^, and Ba^2+^ ions have the same formal charge but different cationic radii, namely 118 pm, 131 pm, and 147 pm, respectively, for nine-fold coordination^34^. The mismatch with the host La cation, whose ionic size is 121 pm, is −2.47%, +8.3% and +21.5%, respectively.

## Results

**Figure 1:**
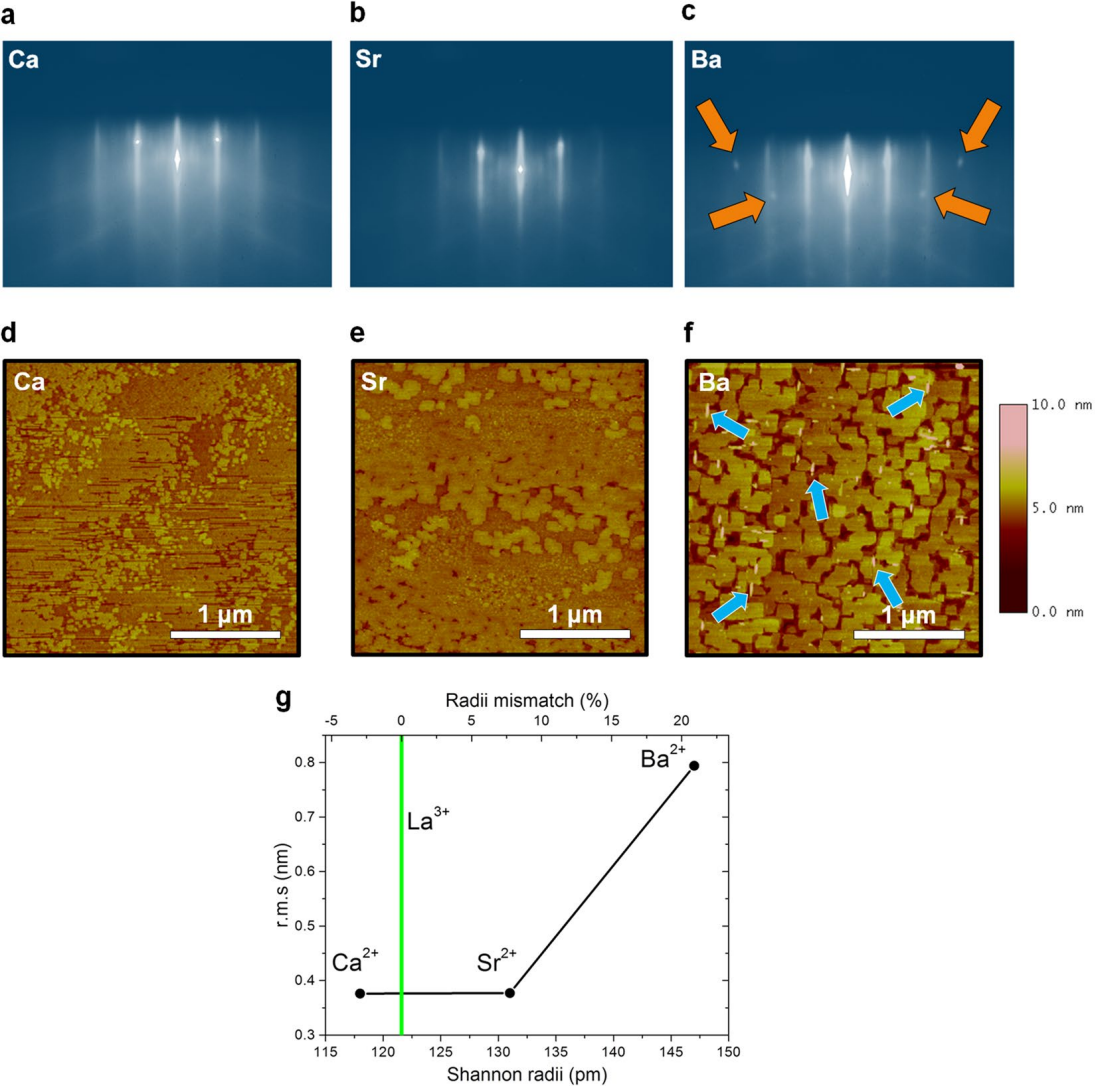
RHEED patterns of the three different bilayers: (**a**) LCCO/LCO, (**b**) LSCO/LCO, (**c**) LBCO/LCO. AFM images of the top surface of the three different bilayers: (**d**) LCCO/LCO, (**e**) LSCO/LCO, (**f**) LBCO/LCO. One can observe the different surface morphology: in particular, in the case of LBCO/LCO, secondary-phase precipitates can be observed (some are marked by blue arrows). The atomic steps with heights of less than one unit cell (u.c.) seen in the figures are attributed to a small substrate miscut angle (~0.1°). (**g**) r.m.s. roughness values of three bilayers versus cation radius (pm).

The growth of each M–I bilayer was monitored by *in-situ* reflection high-energy electron diffraction (RHEED). This surface-sensitive method provides key information about the growth mechanism and rate, the sample quality, and most importantly, can reveal the presence of secondary phase precipitates on the surface of the film during growth. The final RHEED patterns of each bilayer, namely LCCO/LCO, LSCO/LCO, LBCO/LCO, are displayed in Fig. 1a–c, respectively. For Ca- and Sr-doped bilayers, no additional diffraction spots apart from the ones attributed to the typical perovskite-type LCO were observed, which indicates no secondary-phase precipitate formation. However, during the growth of Ba-doped bilayers, such spots were observed as displayed in Fig. 1c. These additional spots are related to the presence of secondary-phase precipitates.

In order to rationalize such findings, we performed a systematic investigation on the surface morphology of each bilayer by means of atomic-force microscopy (AFM). Typical AFM micrographs of three different bilayers are presented in Fig. 1d–f for LCCO/LCO, LSCO/LCO, LBCO/LCO bilayers, respectively. Indeed, these three different bilayers have slightly different surface morphology, i.e. as the dopant size increases from Ca to Ba one can see that the surface becomes visually rougher. This possibly indicates the influence of the dopant on the surface mobility of incoming atoms. Additionally, in the case of the Ba-doped bilayer, one can see relatively high (~3–4 nm) secondary phase precipitates (Supplementary Fig. S3). To quantify the surface roughness, we have used the root mean square (rms) value over the full scanning area. The images demonstrate that Sr-doped and Ca-doped samples have atomically smooth surfaces, whereas the Ba-doped sample is characterized by a remarkably rougher surface. In the case of LCCO/LCO and LSCO/LCO bilayers, the rms roughness was measured as 0.377 nm and 0.376 nm over a scanning area of 6.25 μm^2^. In the case of the LBCO/LCO bilayer, instead, the rms roughness is 0.805 nm, i.e. twice as large over the same scanning area. Most importantly, in the latter, secondary phase precipitates (indicated with blue arrows on Fig. 1f), are detected as already observed by RHEED during the growth. A plot of the root mean square (rms) surface roughness of the bilayers vs. the dopant cation radius (pm) is presented in Fig. 1g.

**Figure 2:**
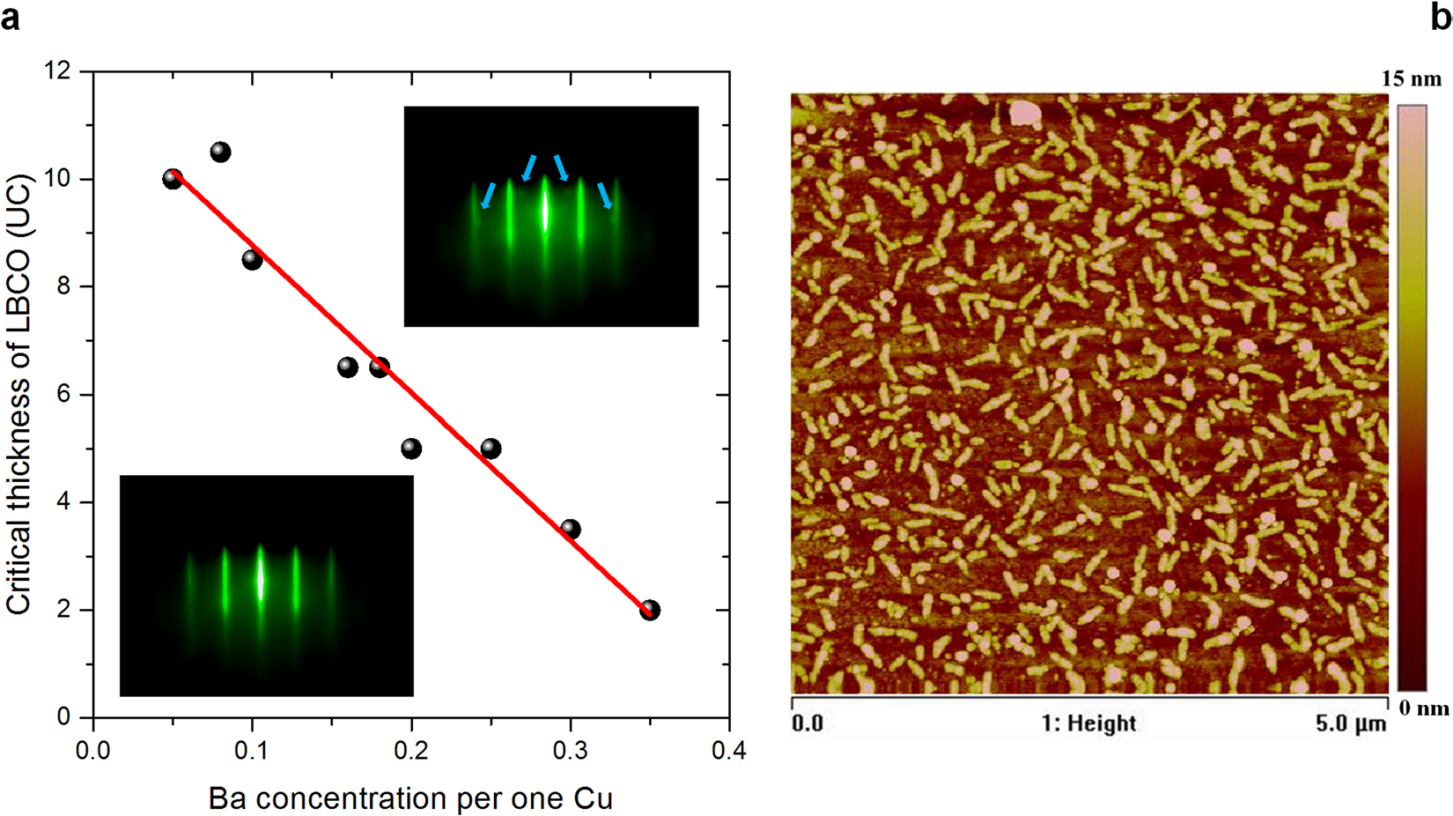
(**a**) Critical thickness of the LBCO film versus Ba concentration at which RHEED patterns indicate nucleation of secondary phase precipitates. The top inset RHEED image has extra diffraction spots in comparison to the bottom RHEED image. (**b**) AFM image of a 15 u.c. thick LBCO (*x* = 0.18) film on LSAO (001) substrate.

Moreover, with the aim of specifically addressing the Ba segregation issue, we have grown more than 50 single-phase Ba-doped LCO epitaxial films with different Ba concentrations in the range from 0.05 to 0.35 on LSAO (001) substrates. None of these films show ideal growth, and are free from formation of secondary phase precipitates. However, the critical thickness at which secondary phase precipitates nucleate decreases linearly as the Ba concentration increases (Fig. 2a). A typical RHEED image of a precipitate-free single-phase LBCO (*x* = 0.18) film with a thickness less than the critical thickness (<5 u.c.) is shown in the bottom inset in Fig. 2a, while the upper inset is a RHEED image of a film thicker than the critical thickness of precipitate formation for this doping level (>6.5 u.c.). Note that diffraction from the LBCO/LCO bilayer exhibits a similar pattern with characteristic spots (cf. Fig. 1). A typical AFM image of the 15 u.c. LBCO single-phase film, in which secondary-phase precipitates are clearly observed, is shown in Fig. 2b. For this particular sample, the rms surface roughness is ~1.74 nm which is twice as large as in an M–I bilayer with a 3 u.c. thick overdoped Ba layer.

Furthermore, by using Auger electron spectroscopy (AES) we confirmed our indirect observations claiming that the precipitates on the surface contain Ba, while the precipitate-free area is the pure La_2_CuO_4_ phase. However, quantifying the chemical composition more precisely is beyond the AES resolution (Supplementary Fig. S4).

**Figure 3:**
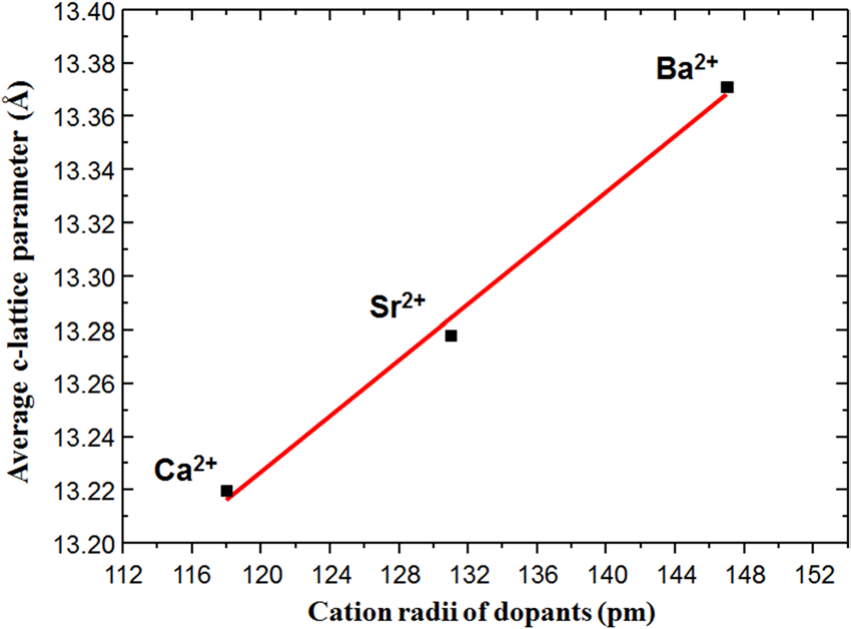
*c*-axis lattice parameter versus ionic radii of dopants in three different bilayers: LCCO/LCO, LSCO/LCO, and LBCO/LCO.

XRD measurements revealed that the shortest *c*-axis lattice constant (13.22 Å) was observed for the LCCO/LCO bilayers, whereas the *c*-axis lattice parameters are 13.28 Å and 13.37 Å for the LSCO/LCO and LBCO/LCO bilayers, respectively. Such findings nicely correlate with the nominal cationic radii in nine fold coordination as illustrated in Fig. 3, as a consequence of Madelung strain.

**Figure 4:**
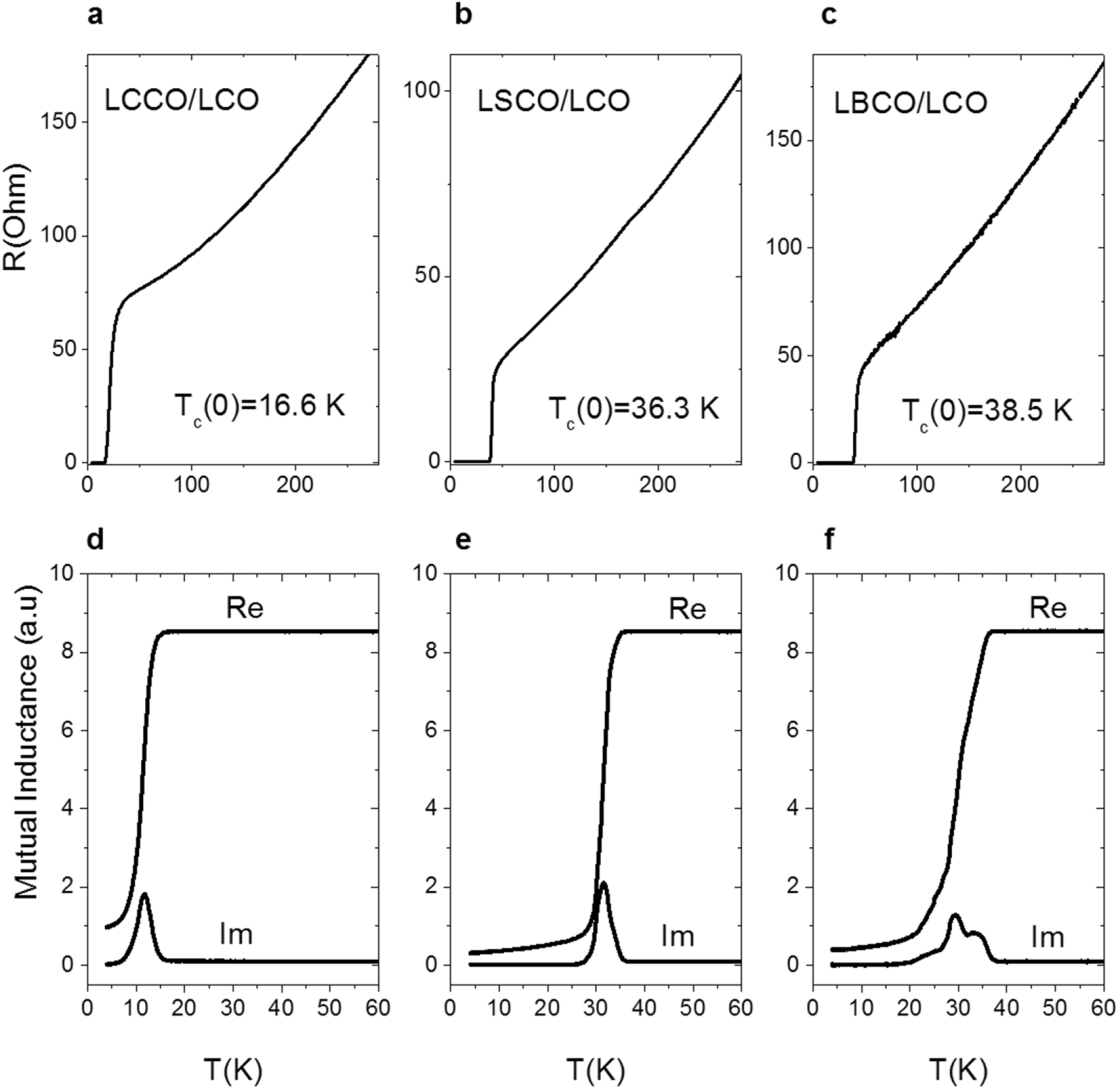
Transport measurements for three different bilayers. The upper panels show resistance versus temperature for (**a**) LCCO/LCO, (**b**) LSCO/LCO, (**c**) LBCO/LCO, whereas the bottom panels show the real (Re) and imaginary (Im) parts of mutual inductance measurements for (**d**) LCCO/LCO, (**e**) LSCO/LCO, (**f**) LBCO/LCO.

DC resistance and magnetic susceptibility measurements in a parallel geometry provided information regarding the superconducting properties of such samples. The most significant data are presented in Fig. 4. In panels a, b, c, the temperature dependence of the electrical resistance is shown for LCCO/LCO, LSCO/LCO, and LBCO/LCO, respectively; the corresponding magnetic susceptibility measurements are displayed in panels d, e, f. The superconducting transition temperature, *T*_c_, was determined from the resistivity measurements as the temperature at which the resistance drops to zero. Notably, these values coincide with the temperatures where the real and the imaginary parts of the mutual inductance signals start to deviate from the normal state values.

**Figure 5:**
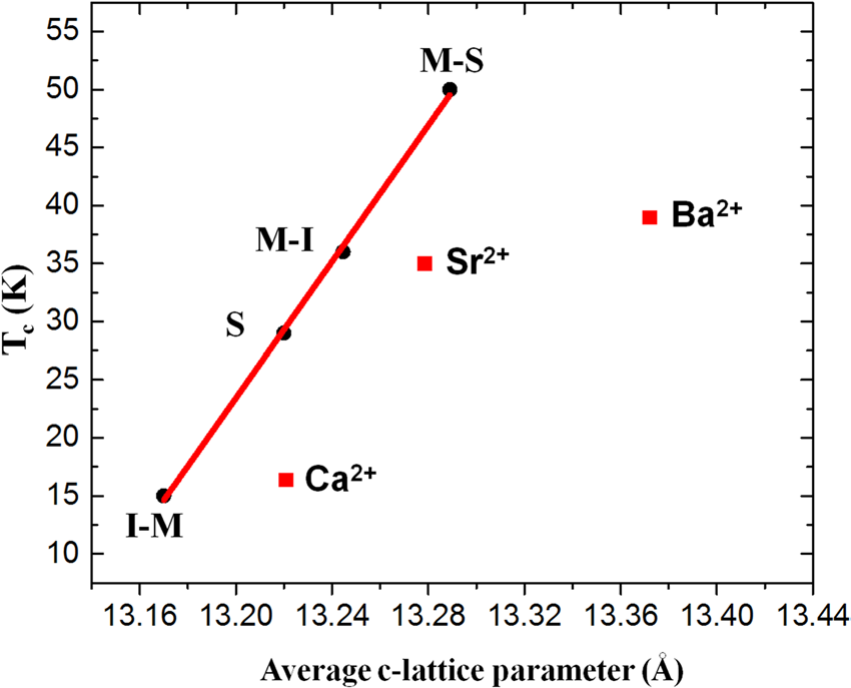
Dependence of *T*_c_ on the average *c*-axis lattice parameter. The red squares show the *T*_c_ values measured in Ca-, Sr-, and Ba-doped bilayers in our study, whereas the black circles refer to  I–M (La_2_CuO_4_/La_1.56_Sr_0.44_CuO_4_),  S (superconducting; La_2_CuO_4+δ_),  M–I (La_1.56_Sr_0.44_CuO_4_/La_2_CuO_4_) and *M-S* (La_1.56_Sr_0.44_CuO_4_/La_2_CuO_4+δ_) structures as reported in ref. 14.

For the LCCO/LCO system, the superconducting critical temperature *T*_c_ is ~17 K, whereas it is ~36 K in the case of the LSCO/LCO bilayer, which is consistent with the literature^13^. For the LBCO/LCO bilayers, a ~10% enhancement of the *T*_c_ value compared to the *T*_c_ for LSCO/LCO bilayers was obtained (*T*_c_ ~ 39 K). Most importantly, the linear correlation between the *c* axis parameter and *T*_c_ as it was proposed in the literature (ref. 14) does not hold in the present case (see Fig. 5).

While the linear extrapolation of *T*_c_ vs *c* data of ref. 14 would lead to a *T*_c_ ~ 70 K (Supplementary Fig. S6), in our case, the measured value is remarkably lower (only ~39 K). In addition, the transition to the superconducting state in the mutual inductance measurements of LBCO/LCO bilayer is broadly ranging from 38.6 K to 20 K with several peaks in the real part, suggesting the presence of regions having different superconducting critical temperatures (Fig. 4f). An overview of the main results including surface roughness, average *c*-axis lattice constant, and *T*_c_ is presented in Table 1. From this, the strong impact of different cations on the superconducting and structural properties of bilayers grown under the same conditions comes to the fore.

**Table 1:** Summary for Ca, Sr, and Ba doped bilayers.

Structure	*T*_c_ (K) (from inductance)	*T*_c_ (K) (from resistance)	Roughness [nm]	*c*-axis parameter [Å]
LCCO/LCO	16.1	16.6	0.377	13.22
LSCO/LCO	35.8	36.3	0.376	13.28
LBCO/LCO	38.1	38.6	0.805	13.37

**Figure 6:**
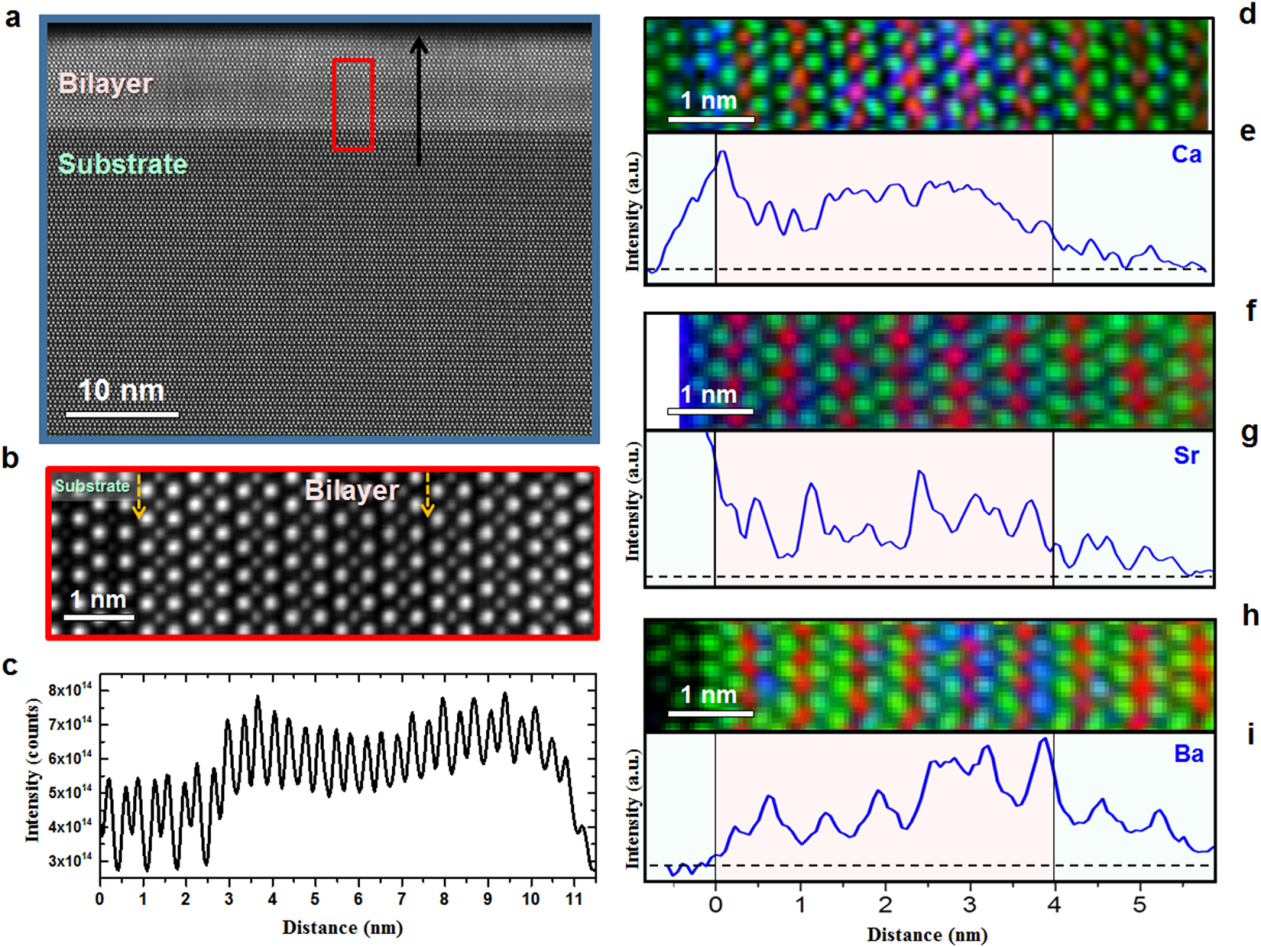
Atomically resolved STEM imaging and EELS spectrum imaging. (**a**) HAADF image showing the growth quality, a defect-free structure and coherent interfaces of LCCO/LCO. (**b**) High magnification of the area highlighted by the red rectangle in a. (**c**) Intensity profile along the black arrow in Fig. 6a, averaged along the horizonta﻿l direction. In d, f, and h, RGB elemental maps (La = green, Cu = red, dopant = blue) are shown. In e, g and i the Ca-, Sr- and Ba-doped bilayers dopant distributions, as obtained from the maps in (**d**,**f**,**h**), are displayed.

In order to gain deeper insight on the interfacial situation and possibly on the discrepancy between the present *c* vs *T*_c_ dependence in comparison with the literature, atomically resolved imaging and spectroscopy were carried out by high-resolution STEM. High-angle annular dark-field (HAADF) images taken from each bilayer proved the growth quality and defect-free structures. Figure 6a depicts the microstructure of the LCCO/LCO bilayer. All atomically resolved HAADF images were taken along the [100] direction of the LSAO substrate. The HAADF image, where the contrast slightly changes in the first two unit cells from the substrate/LCCO bilayer interface, demonstrates the high epitaxial quality of the LCCO/LCO bilayer, a coherent interface, and the absence of extended defects such as misfit dislocations and/or stacking faults. An atomically resolved image at a higher magnification of the highlighted region in Fig. 6a is presented in Fig. 6b. Figure 6c shows the intensity profile of the HAADF image taken from the Ca-doped bilayer (Fig. 6a) along the black arrow, averaged along the horizontal direction. On the HAADF image Ca-doped areas exhibit a darker contrast, while undoped LCO layers appear brighter. Because the HAADF image intensity is approximately proportional to *Z* ^1.7–2^ (where *Z* is the atomic number and *Z*_La_ = 57 and *Z*_Ca_ = 20)^35, 36^, and thus the intensity increase in the first 1–2 unit cells corresponds to the Ca-depleted region, followed by a Ca-doped region with lower intensity. To the best of our knowledge, this is the first example of a successfully grown overdoped LCCO epitaxial layer.

Figure 6d,f and h show RGB (red is Cu, green is La, and blue is dopant) atomic resolution maps of Ca, Sr and Ba doped bilayers as measured by EELS. The average profiles of the dopant distributions obtained from the EELS maps are shown below each RGB map in Fig. 6e,g and i. The nominal interfaces are shown by black lines. To exemplify the base level for the dopant signal observed, the Ba profile averaged from the RGB map of Ba-doped bilayer is presented in Supplementary Fig. S9.

The RGB maps and the average profiles of dopant distributions for Ca-, Sr- and Ba-doped bilayers exhibit characteristic differences. Sr-doped bilayers show the most homogeneous distribution among the dopants [see Fig. 6f and g]. The abruptness of the LSCO/LCO interface can be estimated from the decay of the Sr distribution from the M layer into I layer as ~1.6 ± 0.4 nm, which is in fairly good agreement with the values reported in the literature^10^ for a related system. Conversely, the distribution of Ca and Ba dopants in LCCO/LCO and LBCO/LCO bilayers is less homogeneous. For averaging the distribution lengths from EELS line scans (for each dopant) we averaged several line scans acquired from different regions of the samples.

The atomically resolved EELS RGB map shown in Fig. 6h shows that the Ba concentration increases in the M phase towards the LBCO/LCO interface. This trend is also confirmed by the averaged profile of the Ba dopant presented in Fig. 6i and clearly demonstrates the tendency of Ba to segregate towards the free surface of the film. Most importantly, as a consequence of such Ba migration, the LBCO/LCO interface is quite smeared, i.e. the decay length of Ba ions, obtained as an average from several EELS linescan, is 2.6 ± 0.6 nm, which is considerably larger with respect to the other dopants. Please note that some linescans – as the one displayed in Supplementary Fig. S10 – exhibit a Ba distribution involving the whole nominally undoped area. This may be related to the presence of secondary phase precipitates at the film free surface, which have been observed by RHEED and AFM (see Fig. 1).

As far as the LCCO/LCO bilayer is concerned, we observe a tendency to Ca accumulation at the interface between substrate and the epitaxial layer, followed by a depletion of Ca on the 1^st^ and 2^nd^ unit cells (see Fig. 6a–c) as well as the magnified RGB EELS map and average Ca profile in Fig. 6d and e, respectively. Such an anomalous behavior may be connected with the compressive in-plane strain state in the film and will be the object of further investigations. In this case, the extent of cationic intermixing at the M–I interface can be estimated to be ~1.4 ± 0.4 nm.

## Discussion

The present investigations highlight the profound impact that the choice of the dopant has on the final structural properties of the bilayers, and how this affects the resulting electrical transport properties of the system. In particular, we observe that the out-of-plane *c-*axis lattice parameter as measured by XRD (Supplementary Fig. S2), which is an average over the whole thin-film volume, exhibits a strong dependence on the dopant species, i.e., *c* is proportional to the dopant ionic radius (the larger the dopant radius the larger is the lattice parameter (Fig. 3a). This allows us to tune *c* simply by changing the dopant species while keeping the ionic charge constant. Interestingly, although Ca^2+^ ions are smaller than La^3+^ ions, the Ca-doped bilayer has a larger *c*-axis lattice parameter than pure LCO. This can be explained by the different valences of Ca (2+) and La (3+). Ca^2+^ replaces La^3+^ and maintains the nine-fold coordination. Therefore, Ca^2+^ constitutes a negative charge on the A-site. The electrostatic repulsion of this negative charge with neighboring oxygen ions results in a lattice expansion.

Most importantly, we expect a major impact of the dopant size on the in-plane strain state of the films, in a similar way as was already demonstrated by Lee *et al*. for a related perovskite system^37^. In particular, when Ba^2+^ is employed as a dopant, i.e. in the case of LBCO/LCO bilayers, the maximum in-plane strain is induced. As the HAADF images (see Supplementary Fig. S7) show perfect epitaxial growth of all films without formation of misfit dislocations or other defects which could relieve strain, the only way to obtain strain relaxation in the case of the Ba-doped system is by the rearrangement of dopants within the film, i.e. the segregation of excess Ba towards the film surface. This explains the formation of a secondary phase (likely Ba-based) at the surface^32, 33^, as shown by AFM and RHEED images (Fig. 1c‒f) and revealed by AES (Supplementary Fig. S4). It also explains, why in the case of single-phase films, the critical thickness of precipitate formation during the growth is decreasing with increasing Ba concentrations (see Fig. 2a). The absence of such precipitates in LCCO and LSCO films indicates that Ca^2+^ and Sr^2+^ ions, whose ionic size is more similar to La^3+^ with respect to Ba^2+^, are accommodated in the thin film, i.e. the in-plane strain is maintained. Our results are consistent with those of Lee *et al*.^37^ who found that the smaller size mismatch between the La^3+^ host and dopant cations reduces the segregation level of the dopants. Notably, whereas a homogeneous dopant profile was found in the Sr-doped bilayers, also the Ca-doped bilayers exhibit a certain tendency to inhomogeneity, i.e. Ca^2+^ accumulates towards the substrate possibly as a consequence of the strain state at the substrate–film interface.

Remarkably, we observed a strong deviation from the expected linear dependence of *T*_c_ on the *c*-axis lattice parameter for M–I bilayers, with *T*_c_ of the LBCO/LCO bilayer being lower than expected, while the c-axis lattice parameter is increased. Whereas the linear extrapolation of *T*_c_ vs *c* leads to a predicted *T*_c_~70 K, our measured value is only ~39 K. In order to explain this, we need to consider the dopant distributions at each interface. In particular, the average cationic intermixing extent is as high as 2 u.c. in the case of LBCO/LCO bilayers (the wide Ba distribution is also detected by EELS line scans (see Supplementary Fig. S10)). We believe that the anomalous Ba redistribution is a consequence of Ba segregation towards the film surface, which eventually results in a particularly smeared M‒I interface. Such a finding can account for the reduced *T*_c_ for the LBCO/LCO bilayers as demonstrated recently for a related LCO-based system for which a smeared interface leads to a classical doping mode, the so-called “homogeneous doping”^38^, in which, at equilibrium, the hole concentration is increased in correspondence to the randomly distributed ionic dopant point defects, rather than to a striking interface effect, and defines the final local physical properties. In such a situation (only homogeneous doping is active), one expects *T*_c_ not to exceed the values which are normally obtained in doped single-phase systems, i.e. max *T*_c_ ~ 40 K for optimally doped LBCO samples epitaxially grown on LaSrAlO_4_ (001) substrates^29^.

For both systems, although a certain dopant redistribution is present at the interface, we believe that the behavior is consistent with the typical HT-IS. Notably, in the case of the LSCO/LCO interface as investigated by Gozar *et al*.^10, 16^, Sr spread into the nominally undoped phase for about 1 u.c., i.e. 1.3 nm, in agreement with our observations on both LCCO/LCO and LSCO/LCO structures. Nonetheless, our findings allow us to undoubtedly ascribe HT-IS to an electronic effect, rather than to ionic doping. Therefore, in the present work, the reduced *T*_c_ of the LCCO/LCO interface (the dopant spread beyond the interface is 1.4 nm), may be linked to the small *c*-axis parameter, in agreement with the linear *c*-vs-*T*_c_ relation. The Sr-doped bilayers show consistency with the previous studies not only in *T*_*c*_ but also in the Sr redistribution length^10^.

Lastly, a small systematic shift of Sr- and Ca-doped bilayers’ *c*-lattice parameters (see Fig. 4) compared to the literature data was observed. This could be explained by the difference in the layer thicknesses, since the layers grown in ref. 14 are 20 u.c. thick, whereas in our case they are just 3 u.c. thick, thus being possibly affected by a different in-plane strain state.

## Conclusions

In conclusion, we used a number of complementary experimental methods, including high-resolution XRD, AFM, transport measurements and spherical aberration corrected STEM-EELS, in order to study high-temperature superconducting interfaces in La_1.6_A_0.4_CuO_4_/La_2_CuO_4_ bilayers grown by atomic-layer-by-layer oxide MBE, where A = Ca^2+^, Sr^2+^, and Ba^2+^. We found that the *c*-axis lattice parameter increases linearly with the dopant size. Surprisingly, *T*_c_ was found to depend non-linearly on the *c*-axis lattice constant and saturates at about 40 K, whereas *T*_c_ was expected to rise up to about 70 K for the LBCO/LCO case due to the interplay between hole leakage and Madelung strain. This is assigned to a different redistribution of the dopant ions across the interface. In particular, as a consequence of the large ionic size mismatch between the dopant and the host cation, Ba segregation occurs and gives rise to a remarkably smeared interface. In the case of LCCO/LCO and LSCO/LCO systems, the interfaces were found to be sharper (yet not atomically sharp). As a consequence of such a different interface structure, distinct phenomena occur for inducing interface superconductivity: in the LCCO/LCO and LSCO/LCO cases, striking interface effects, i.e. electronic redistribution, are predominant, whereas, in the case of LBCO/LCO, HTSC is rather ascribed to “classical” homogeneous doping determined by cationic intermixing. In such a “conventional” situation, the expected *T*_c_ enhancement due to the interface effect is prevented.

This work highlights the profound impact that ionic intermixing may have on the definition of the final properties of oxide epitaxial interfaces and demonstrates that future studies of ionic effects at interfaces, in particular on cationic redistribution during growth, are of paramount importance for the full understanding of such structures.

## Methods

### ALL-oxide MBE growth

La_1.6_A_0.4_CuO_4_ (metallic)-La_2_CuO_4_ (insulating) (A = Ca^2+^, Sr^2+^, Ba^2+^) bilayers consisting of 3 unit cells of the overdoped and 3 unit cells of the undoped LCO layer were grown on LaSrAlO_4_ (001) (LSAO) substrates (Crystec GmbH) using atomic-layer-by-layer oxide MBE (DCA Instruments). The doping concentration of the metallic overdoped layer was intentionally x = 0.4 to avoid superconductivity in the M layer. The total thickness was kept constant and the thickness of individual layers was 3 u.c. each to provide a reasonable comparison with the analogous Sr-based bilayers studied in ref. 13. The deposition conditions used for growing the samples were *T*_s_ = 600 °C (pyrometer reading) at a pressure of ~3·10^−5^ Torr (mixed ozone, radical oxygen and molecular oxygen atmosphere). After bilayer growth, all samples were cooled in vacuum, from *T*_s_ = 210 °C to room temperature, in order to exclude any influence on the electrical properties from interstitial oxygen doping (see ref. 30 for further information). During the growth, reflection high-energy electron diffraction (RHEED) was used to control the sample quality.

### Conductivity measurements and structural characterization

Electrical measurements in a Van der Pauw (four-point-probe) configuration with alternative DC currents of ±20 μA were employed. Simultaneously, measurements of the imaginary and the real parts of the mutual inductance *M*(*T*) were carried out by magnetic susceptibility measurements in a two-coil configuration (parallel geometry) with an AC current of 50 μA at a frequency of 1 kHz. The temperature was varied from room temperature to 4.2 K (liquid helium) using a motorized custom-designed dipstick (*T* change rate <0.1 K/s). Surface morphology, crystal structure characterizations, and AES investigations (at 10 kV) were performed by atomic force microscopy (AFM) (Nanoscope III) and high-resolution X-ray diffraction (XRD) (Bruker D8 Cu-Kα1 = 1.5406 Å), JEOL JAMP-7810 Auger microprobe, respectively.

### Scanning transmission electron microscopy

For representative cross-sectional electron transparent samples, standard sample preparation procedure including mechanical grinding, tripod wedge polishing and argon ion milling with a liquid nitrogen cooled stage was performed. For argon ion thinning, a precision ion polishing system (PIPS II, Model 695) was used at low temperature. For all STEM analyses, a probe-aberration-corrected JEOL JEM-ARM200F STEM equipped with a cold field-emission electron source, a probe *C*_s_-corrector (DCOR, CEOS GmbH), a large solid-angle JEOL Centurio SDD-type energy-dispersive X-ray spectroscopy (EDXS) detector, and a Gatan GIF Quantum ERS spectrometer was used. STEM imaging and both EDXS and electron energy-loss spectroscopy (EELS) analyses were performed at probe semi-convergence angles of 20 mrad and 28 mrad, resulting in probe sizes of 0.8 Å and 1.0 Å, respectively. The collection angle range for high-angle annular dark-field (HAADF) images was 75–310 mrad. A collection semi-angle of 111 mrad was used for EELS investigations. EEL spectrum images were processed using the multivariate weighted principal component analysis routine (PCA) (MSA Plugin in Digital Micrograph) developed by M. Watanabe^39^ to reduce the noise in the data. In order to separate overlapping edges in each spectrum, such as La-M_5,4_, Cu-L_3,2_ and Ba-M_5,4_ in our case, the multiple linear least square fitting (MLLS)^40^ was used. For overlapped signals, MLLS fitting windows of 650–1100 eV for Ba-M_5,4_, La-M_5,4_, Cu-L_3,2_, 305–390 eV for Ca-L_3,2_, and 1750–2100 eV for Sr-L_3,2_ edges were used. The integration windows used for Ca-L_3,2_, Ba-M_5,4_, La-M_5,4_, Cu-L_3,2_, Sr-L_3,2_, and edges are 343–394 eV, 772–815 eV, 822–868 eV, 935–961 eV, 1935–2066 eV, respectively.

## Electronic supplementary material

Supplementary Info

## References

[1] Hwang HY (2012). Emergent phenomena at oxide interfaces. Nat. Mater..

[2] Mannhart J, Schlom DG (2010). Oxide Interface–An Opportunity for Electronics. Science.

[3] Ahn CH, Triscone J-M, Mannhart J (2003). Electric field effect in correlated oxide systems. Nature.

[4] Ohtomo A, Hwang HY (2004). A high-mobility electron gas at the LaAlO_3_/SrTiO_3_ heterointerface. Nature.

[5] Reyren N (2007). Superconducting Interfaces Between Insulating Oxides. Science.

[6] Maier J (2005). Nanoionics: ion transport and electrochemical storage in confined systems. Nat. Mater..

[7] Tsukazaki A (2007). Quantum Hall Effect in Polar Oxide Heterostructures. Science.

[8] Brinkman A (2007). Magnetic effects at the interface between non-magnetic oxides. Nat. Mater..

[9] Biscaras J (2010). Two-dimensional superconductivity at a Mott insulator/band insulator interface LaTiO_3_/SrTiO_3_. Nat. Commun..

[10] Gozar A (2008). High-temperature interface superconductivity between metallic and insulating copper oxides. Nature.

[11] Logvenov G (2008). Engineering interfaces in cuprate superconductors. Phys. B Condens. Matter.

[12] Loktev VM, Pogorelov YG (2008). Model for modulated electronic configurations in selectively doped multilayered La_2_CuO_4_ nanostructures. Phys. Rev. B.

[13] Logvenov G, Gozar A, Bozovic I (2009). High-Temperature Superconductivity in a Single Copper-Oxygen Plane. Science.

[14] Butko VY, Logvenov G, Božović N, Radović Z, Božović I (2009). Madelung Strain in Cuprate Superconductors – A Route to Enhancement of the Critical Temperature. Adv. Mater..

[15] Zhou H (2010). Anomalous expansion of the copper-apical-oxygen distance in superconducting cuprate bilayers. Proc. Natl. Acad. Sci.

[16] Smadici S (2009). Superconducting Transition at 38 K in Insulating-Overdoped La_2_CuO_4_-La_1.64_Sr_0.36_CuO_4_ Superlattices: Evidence for Interface Electronic Redistribution from Resonant Soft X-Ray Scattering. Phys. Rev. Lett..

[17] Suter A (2011). Two-Dimensional Magnetic and Superconducting Phases in Metal-Insulator La_2−x_Sr_x_CuO_4_ Superlattices Measured by Muon-Spin Rotation. Phys. Rev. Lett..

[18] Stilp E (2013). Magnetic phase diagram of low-doped La_2−x_Sr_x_CuO_4_ thin films studied by low-energy muon-spin rotation. Phys. Rev. B.

[19] Yacoby Y, Zhou H, Pindak R, Božović I (2013). Atomic-layer synthesis and imaging uncover broken inversion symmetry in La_2−x_Sr_x_CuO_4_ films. Phys. Rev. B.

[20] Wu J (2013). Anomalous independence of interface superconductivity from carrier density. Nat. Mater..

[21] Gasparov VA, Božović I (2015). Complex conductance of ultrathin La_2−x_Sr_x_CuO_4_ films and heterostructures. Low Temp. Phys..

[22] Panagopoulos C, Majoros M, Nishizaki T, Iwasaki H (2006). Weak Magnetic Order in the Normal State of the High-Tc Superconductor La_2−x_Sr_x_CuO_4_. Phys. Rev. Lett..

[23] Logvenov G (2010). Comprehensive study of high-Tc interface superconductivity. J. Phys. Chem. Solids.

[24] Bollinger AT (2011). Superconductor-insulator transition in La_2−x_Sr_x_CuO_4_ at the pair quantum resistance. Nature.

[25] Pereiro J (2012). Insights from the study of high-temperature interface superconductivity. Philos. Trans. R. Soc. Lond. Math. Phys. Eng. Sci..

[26] Logvenov G, Gozar A, Bozovic I (2013). High Temperature Interface Superconductivity. J. Supercond. Nov. Magn..

[27] Bozovic I, Ahn C (2014). A new frontier for superconductivity. Nat. Phys.

[28] Gozar A, Bozovic I (2016). High temperature interface superconductivity. Phys. C Supercond. Its Appl.

[29] Sato H, Tsukada A, Naito M, Matsuda A (2000). Absence of 1/8 anomaly in strained thin films of La_2−x_Ba_x_CuO_4_. Phys. Rev. B.

[30] Baiutti F, Cristiani G, Logvenov G (2014). Towards precise defect control in layered oxide structures by using oxide molecular beam epitaxy. Beilstein J Nanotechnol.

[31] Bozovic I, Logvenov G, Belca I, Narimbetov B, Sveklo I (2002). Epitaxial Strain and Superconductivity in La_2−x_Sr_x_CuO_4_ Thin Films. Phys. Rev. Lett..

[32] Moodenbaugh AR, Sabatini RL, Xu Y, Ochab J, Huber JG (1992). Solubility of Ca in superconducting La_2−x_Ca_x_CuO_4_. Phys. C Supercond.

[33] Shen L, Salvador P, Mason TO, Fueki K (1996). High temperature electrical properties and defect chemistry of La_2−x_Ca_x_CuO_4−y_ superconductors—II. Defect structure modeling. J. Phys. Chem. Solids.

[34] Shannon RD (1976). Revised effective ionic radii and systematic studies of interatomic distances in halides and chalcogenides. Acta Crystallogr. Sect. A.

[35] Pennycook SJ, Jesson DE (1990). High-resolution incoherent imaging of crystals. Phys. Rev. Lett..

[36] Wang Y (2016). Atomic-Scale Quantitative Analysis of Lattice Distortions at Interfaces of Two-Dimensionally Sr-Doped La_2_CuO_4_ Superlattices. ACS Appl. Mater. Interfaces.

[37] Lee W, Han JW, Chen Y, Cai Z, Yildiz B (2013). Cation Size Mismatch and Charge Interactions Drive Dopant Segregation at the Surfaces of Manganite Perovskites. J. Am. Chem. Soc..

[38] Baiutti F (2015). High-temperature superconductivity in space-charge regions of lanthanum cuprate induced by two-dimensional doping. Nat. Commun..

[39] Bosman M, Watanabe M, Alexander DTL, Keast VJ (2006). Mapping chemical and bonding information using multivariate analysis of electron energy-loss spectrum images. Ultramicroscopy.

[40] The use of MLLS fitting approach to resolve overlapping edges in the EELS spectrum at the atomic level|Gatan, Inc. Available at: http://www.gatan.com/use-mlls-fitting-approach-resolve-overlapping-edges-eels-spectrum-atomic-level.

