# Supplementary material for: Dopant size effects on novel functionalities: High-temperature interfacial superconductivity
Source: Sci Rep. 2017 Mar 28;7:453. doi: 10.1038/s41598-017-00539-4 (PMC5428683; doi:10.1038/s41598-017-00539-4)
Supplement: Supplementary file 1 — Supplementary Info [file 41598_2017_539_MOESM1_ESM.pdf]

Supplementary Information

Dopant size effects on novel functionalities: High-temperature interfacial superconductivity

Y. Eren Suyolcu[1,], Yi Wang[1], Federico Baiutti[1], Ameer Al-Temimy[1,2], Giuliano Gregori[1], Georg Cristiani[1], Wilfried Sigle[1], Joachim Maier[1], Peter A. van Aken[1], Gennady Logvenov[1]*

[1]Max Planck Institute for Solid State Research, Heisenbergstrasse 1, 70569, Stuttgart, Germany

[2]Al-Nahrain Nanorenewable Energy Research Center, Al-Nahrain University, Baghdad, Iraq

y.e.suyolcu@fkf.mpg.de

This file contains:
Supplementary Figures S1-S10 and Supplementary Notes

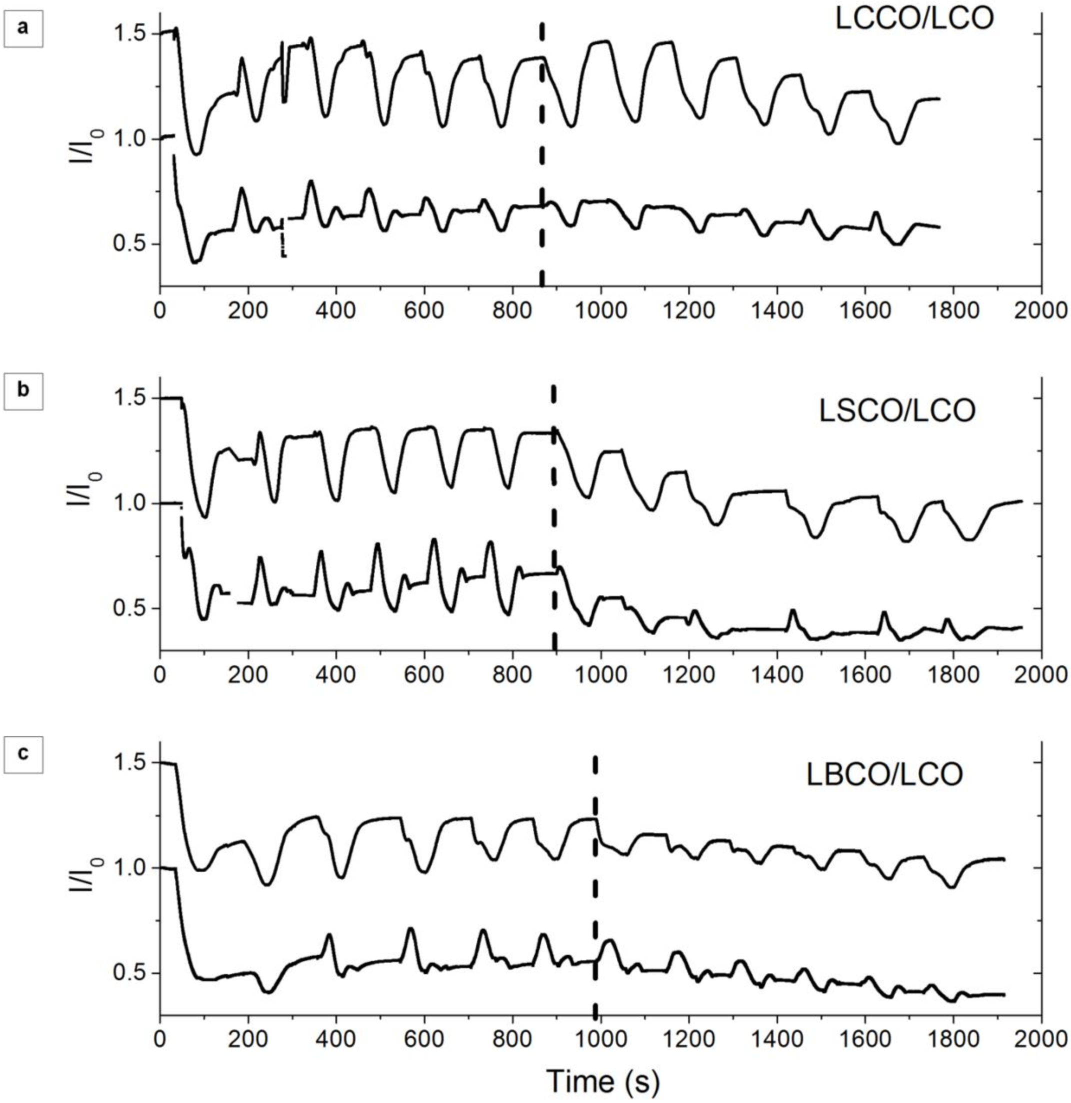

Supplementary Figure S1. RHEED oscillations observed during the growth of LCCO/LCO, LSCO/LCO, and LBCO/LCO bilayers. The intensities were integrated over selected areas, and one oscillation corresponds to a half u.c. thickness of the La_2CuO_4-based crystal structure.

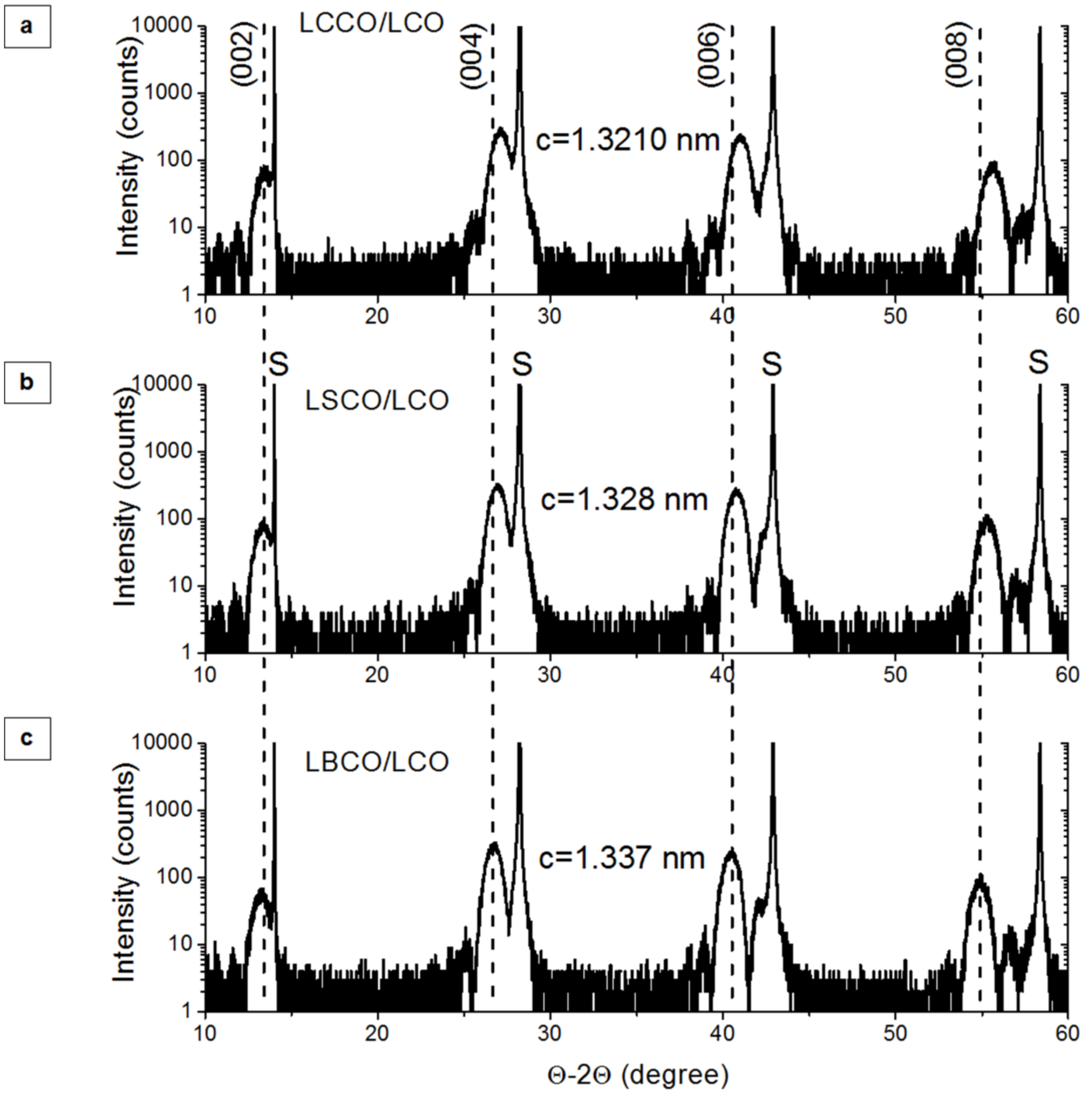

Supplementary Figure S2. XRD θ-2θ scans for three different bilayers: a) LCCO/LCO, b) LSCO/LCO, and c) LBCO/LCO.

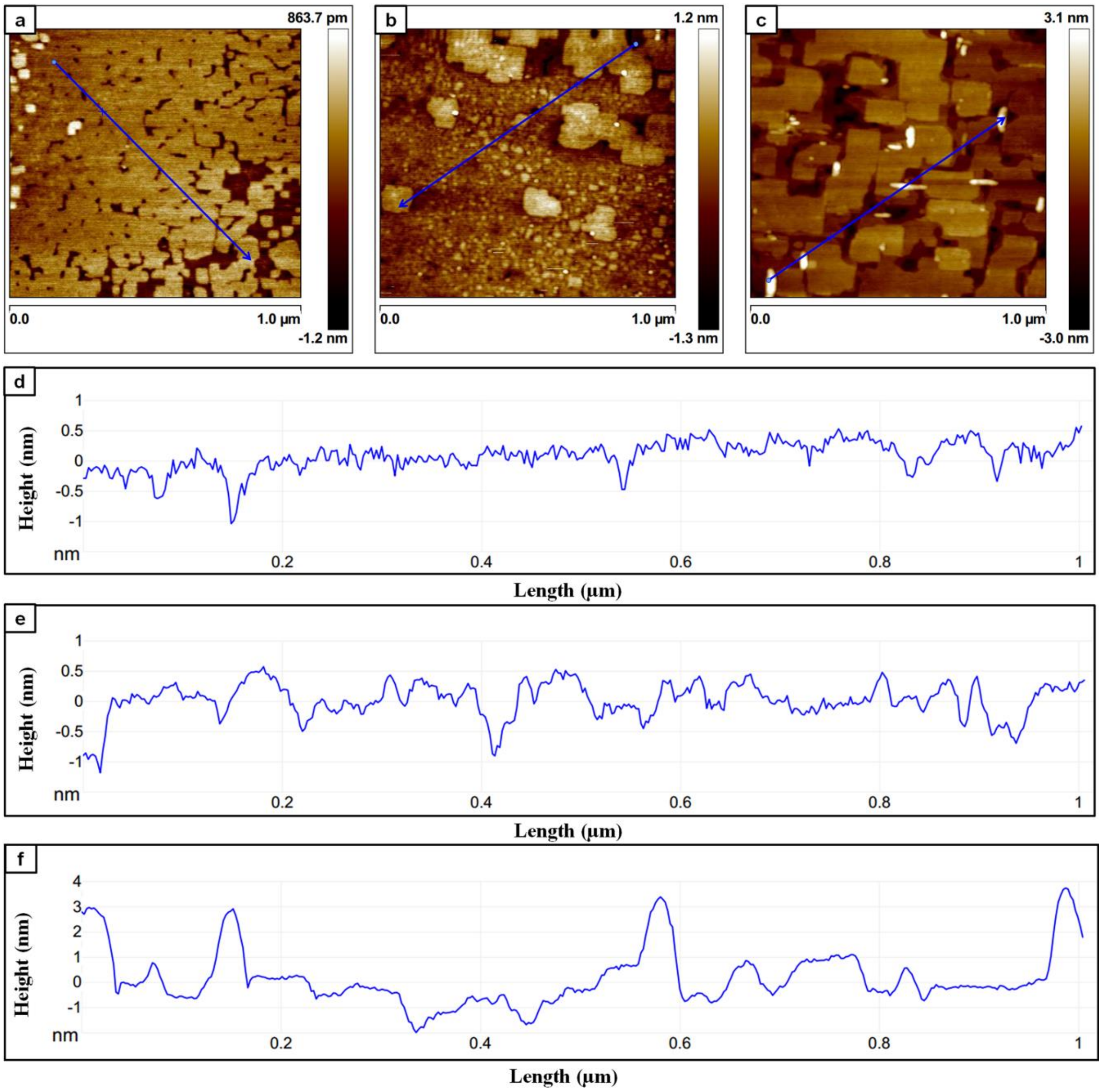

Supplementary Figure S3. AFM images of three different bilayers: a) LCCO/LCO, b) LSCO/LCO, c) LBCO/LCO. Line scan profiles obtained from the AFM images given in a-c: d) LCCO/LCO, e) LSCO/LCO, f) LBCO/LCO. One can observe the different surface morphology. In the case of LBCO/LCO, secondary phase precipitations can be observed with up to 3-4 nm height.

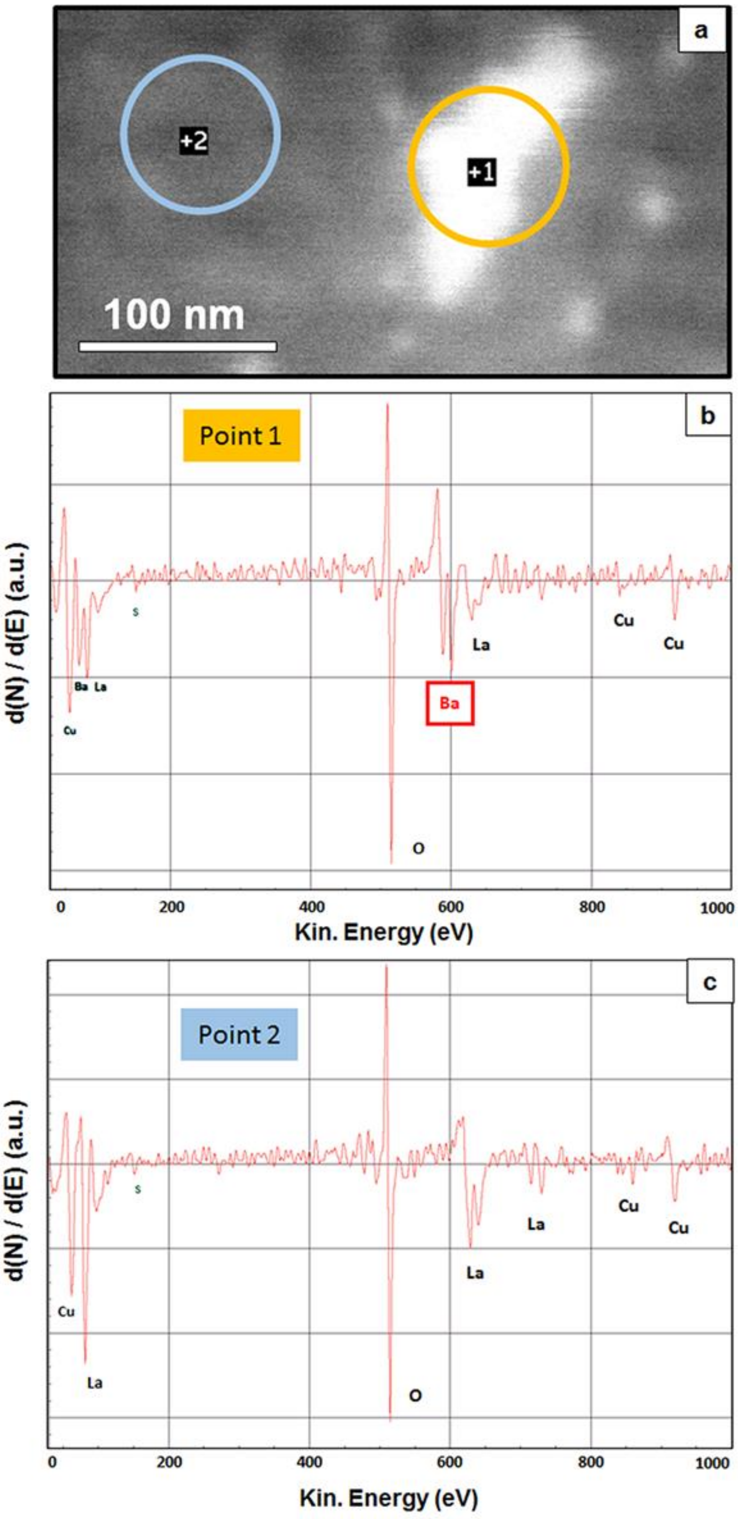

Supplementary Figure S4. Secondary electron image and Auger spectra of the Ba-doped bilayer in the kinetic energy ranges 0-1000 eV. a) secondary electron image of the surface, and selected point AES spectra taken from b) point 1 (from precipitation) and c) point 2 (precipitation-free area) proving the Ba-enrichment at the precipitations on the surface.

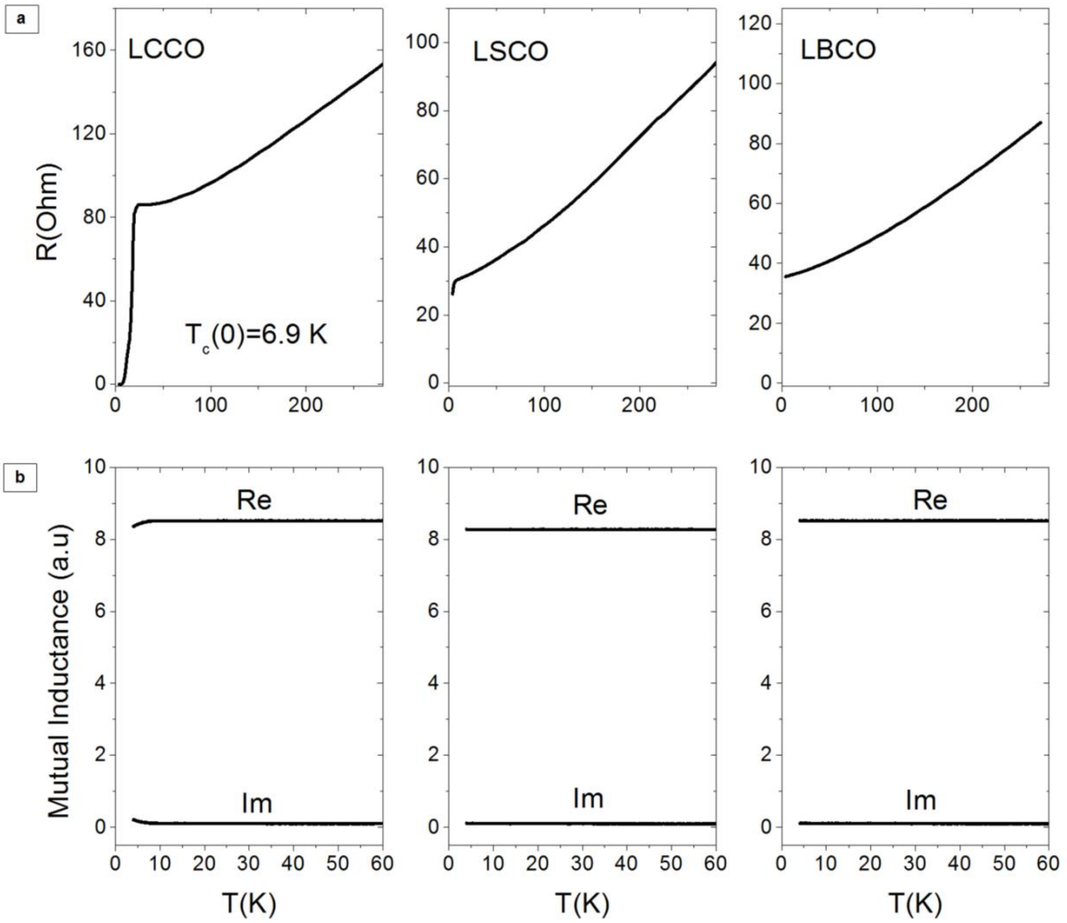

Supplementary Figure S5. Transport measurements versus temperature for three representative overdoped layers. a) The upper row shows resistance versus temperature: LCCO (left), LSCO (center), LBCO (right), b) the bottom row shows the imaginary and real parts of the mutual inductance measurements: LCCO (left), LSCO (center), LBCO (right).

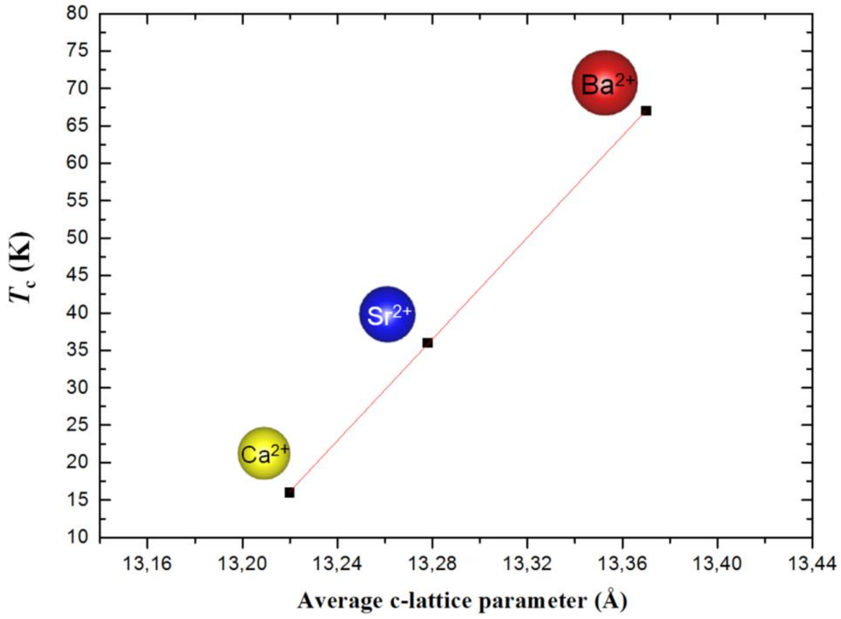

Supplementary Figure S6. Extrapolated dependence of the superconducting critical temperature, T_c, on the average *c*-axis lattice parameters. Black data points show the linear extrapolated T_c value for the Ba-doped bilayer as well as the measured T_c values for Ca- and Sr-doped bilayers in our study.

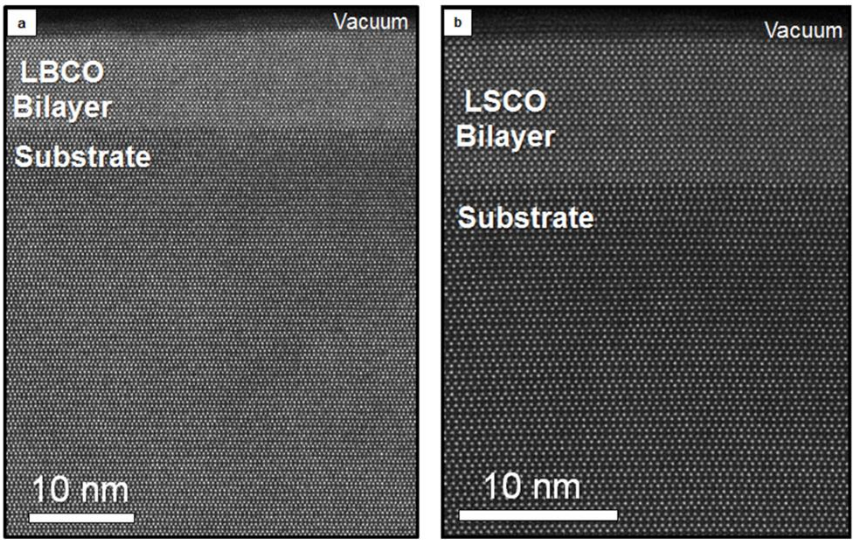

Supplementary Figure S7. Low magnification STEM-HAADF images of a) the LBCO/LCO bilayer and b) the LSCO/LCO bilayer.

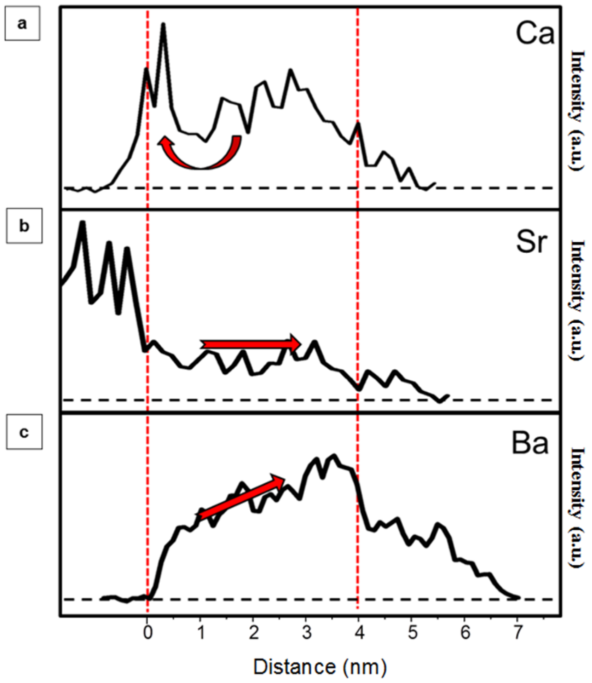

Supplementary Figure S8. Energy-dispersive X-ray spectroscopy (EDXS) line scan profiles across the interfaces, showing dopant distribution of each dopant: a) Ca, b) Sr and c) Ba. Red dashed lines indicate the nominal interfaces and black dashed lines indicate zero intensity.

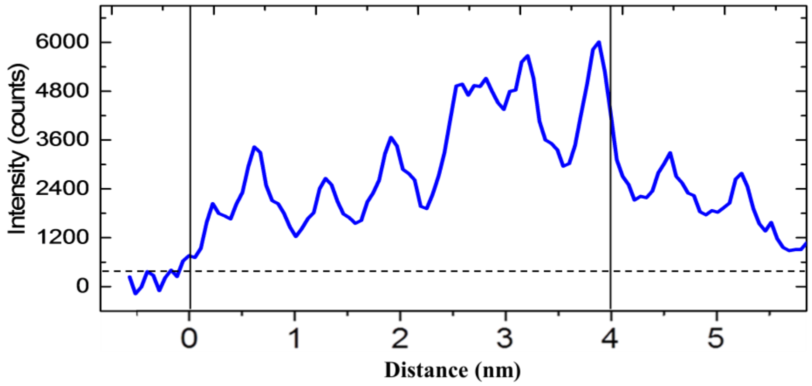

Supplementary Figure S9. Ba-doped bilayer's dopant distribution as a plot of counts vs. distance obtained from the map in Figure 6h. Black lines indicate the nominal interfaces and dashed lines indicate zero intensity.

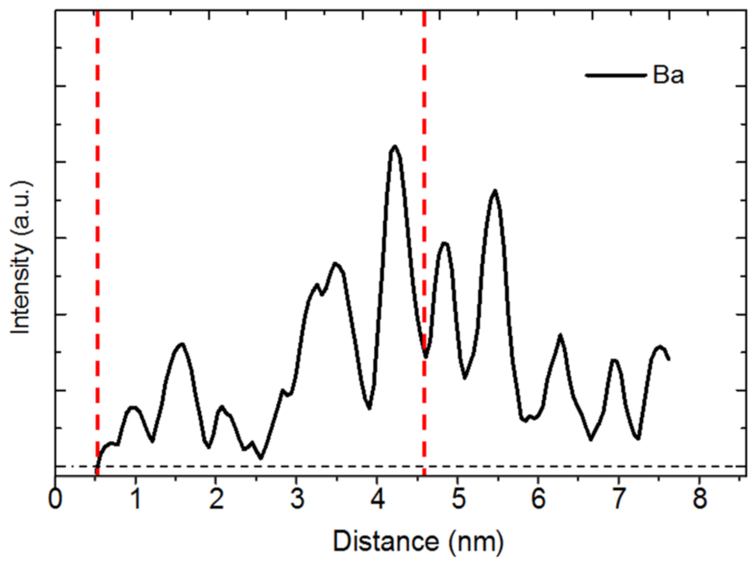

Supplementary Figure S10. Ba EELS line scan profile showing not only inhomogeneous dopant distribution but also the long diffusion length (for this specific line scan, up to ~7.5nm). Red dashed lines indicate the nominal interfaces and black dashed lines indicate zero intensity.

Supplementary Notes

In-situ characterization

The typical RHEED intensity oscillations of the specular spot and the first Bragg line (01), for the growth of three different bilayers namely LSCO/LCO, LBCO/LCO, and LCCO/LCO are plotted as a function of time in Supplementary Fig. S1.

Structural characterization

From the position of Bragg peaks (00*l*), where l = 2, 4, 6 and 8, the average *c*-axis lattice parameter for each bilayer was calculated. XRD Θ–2Θ diffraction scans for three different bilayers, showing a single peak for the two phases in a similar way to what was reported in Ref. 14 and in agreement with the "Madelung strain" situation, are presented in Supplementary Fig. S2.

Auger Electron Spectroscopy

For further understanding of the precipitations on the sample surface (i.e. Ba-doped bilayer) AES investigations were performed (at 10 kV) and revealed Ba-rich precipitations on the surfaces. Corresponding secondary electron images and spectra are presented in Supplementary Fig. S3.

Ca- , Sr-, Ba-doped single phase thin films

For a further comparison, additional Ca-, Sr-, and Ba-doped single phase layers were grown on LSAO substrates and transport measurements were performed for each. The Sr- and Ba-overdoped (x= 0.4) layers in the bilayers systems were not superconducting by their own, whereas the Ca-overdoped (x=0.4) single layer shows a superconducting transition at low temperatures T_c ~ 6.9 K (see Supplementary Fig. S3).

Further STEM investigations

The low magnification HAADF images of LBCO/LCO and LSCO/LCO bilayers reveal that no extended defects are present in none of the samples (Supplementary Fig.S7). EDXS

investigations were performed together with the EELS analyses and EDXS line scan profiles of the three bilayers are presented in Supplementary Fig. S8. EDXS line scan profiles are consistent with EELS data. Ba-doped bilayer's dopant distribution as a plot of counts vs. distance obtained from the map in Figure 6h in the manuscript is shown in Supplementary Fig. 9. A separate EELS line scan obtained from LBCO/LCO bilayer showing the wide Ba distribution is presented in Supplementary Fig. S10.
